# Physical examination checklist for medical students: can less be more?

**DOI:** 10.5116/ijme.591b.3022

**Published:** 2017-06-12

**Authors:** Mohammed Elhassan

**Affiliations:** 1 UCSF Fresno Center for Medical Education and Research, Fresno, CA, USA

**Keywords:** Physical examination, medical students, rational clinical examination, evidence-based physical diagnosis, physical diagnosis secrets

## Introduction

As part of their training, medical students and postgraduates are usually required to demonstrate competency in conducting physical examination skills for graduation and advancement.  This is typically done by observing them perform a checklist of physical examination maneuvers in real or standardized patients. It is important that medical educators emphasize the importance of the art of physical examination to their learners and encourage them to master it. Sir William Osler once said: “Observe, record, tabulate, communicate. Use your five senses. Learn to see, learn to hear, learn to feel, learn to smell, and know that by practice alone you can become expert.” Nevertheless, a question is often asked by medical students and residents: are all the physical examination skills traditionally taught in medical schools as important now as they used to be in the past?

As I tried to explain in another paper,^1^ physical examination skills are essential for good medical care even in the presence of the advanced diagnostic surrogates. Yet, medical educators now need to reconsider the physical examination checklist that they use to assess the physical examination skills of their learners. The contemporary checklist should emphasize maneuvers that are useful in terms of their sensitivity, specificity, positive likelihood ratio (LR+), and negative likelihood ratio (LR-) and exclude those which no longer provide such diagnostic strength. In my own teaching, I do not criticize my learners if they omit or de-emphasize certain exam maneuvers during their presentation since the data argues that these maneuvers are not statistically useful or can be replaced by other better examination tests. Here are some examples of such maneuvers that are commonly taught by medical educators.

### Tactile vocal fremitus and vocal resonance

Tactile vocal fremitus (TVF) is the vibration sensation felt by the examining hand during palpation of the patient’s chest wall (specifically the intercostal spaces) while the patient speaks (e.g. saying “toys for tots” or “ninety-nine”). It is commonly performed as part of the chest examination to detect pathologies such as pneumothorax or pleural effusion (decreased TVF) or consolidation (increased TVF). However, similar findings can be elicited by listening with a stethoscope to vocal resonance (VR) during lung examination. In one study using hospitalized patients with respiratory symptoms, TVF yielded likelihood ratio (LR) findings that were similar to VR for detecting pleural effusion (has LR+ of 5.7 versus 6.5 when diminished and LR- of  0.2 versus 0.3 when normal, for TVF versus VR respectively).^2^ Additionally, TVF has been found to be clinically useful only in patients with pleural effusion.  In other words, one could omit TVF during lung exam and instead listen to VR while auscultating lung fields to note for any asymmetry. Listening with the stethoscope is less subjective than palpating with hands and beginners in physical exam training might find VR easier to interpret and more practical than TVF. Also, a busy medical resident probably will prefer not to spend time doing a second examination test to elicit the same finding. Thus, TVF could be abandoned.

### The liver span

The liver span is performed by percussion of the right hemothorax together with palpation of the right upper abdominal quadrant and is used to estimate liver size at the bedside. It is usually taught as part of the examination of the abdomen. However, this technique has been found to have non-significant LRs.^3^ Also, liver size is often underestimated by this method and physicians vary in their interpretation of their findings.^4^ In most cases when liver pathology is suspected, imaging (at least an ultrasound) of the liver will still be required to better delineate the size and textures of the liver to make a proper differential diagnosis. Frequently, I encounter learners doing or commenting on liver span during standardized testing scenarios but I cannot remember a clinical encounter with real patients where I really wanted the medical student or resident to tell me his/her finding about liver span.  Therefore, Liver span could also be dropped from the list of physical exam skills that need to be taught.

### Kernig's sign and Brudzinski's sign for meningitis

Kernig's sign and Brudzinski's sign are popular among medical students in physical exam evaluation of patients with fever and headache, a common reason for hospital admission worldwide, especially when meningitis is in the differential diagnosis. However, in 190 patients admitted with acute encephalitis syndrome^5^ and 297 patients with suspected meningitis,^6^ these two signs performed poorly in discriminating between patients with or without meningitis as determined by subsequent cerebral spinal fluid analysis, and their LRs were close to 1. So, in clinical practice they may be of little help in clinical decision making and, given the discomfort they can cause for patients, they can probably be dropped from the clinical exam checklist, as well.

### Homans sign for deep venous thrombosis

Homans sign for deep venous thrombosis (DVT) also seems well-known among medical students who tend to mention it during presentation of cases of pulmonary embolism or DVT. Homans sign, or the dorsiflexion sign, is elicited by forceful dorsiflexion of the leg and noting whether there is increased resistance (or calf pain, although some authors claim that pain was not described in the original paper by Homans).  A positive test was thought to indicate DVT, but clinical utility was found to be doubtful with non-significant LRs.^7^^,^^8^ Other validated scoring systems that rely on other lower extremity exam findings (e.g. Wells scoring system for DVT) are widely available and more reliable in making the diagnosis. Therefore, and since it may be associated with patient discomfort and theoretical risk of dislodging an existing clot, physicians should discourage learners from attempting to elicit this sign during lower extremity examination of patients suspected to have DVT.

## Conclusions

In the age of evidence-based medicine, teachers of physical examination skills in medical schools and postgraduate training programs should incorporate more of what we now know about the validity and usefulness of the various physical examination maneuvers in reaching a diagnosis. Tests sensitivities, specificities, LRs, and how these measures affect the post-test probabilities should be emphasized in courses and curricula pertaining to physical examination skills. Medical students should be encouraged and instructed to look into the many resources that are now available in medical literature which tackle this topic, such as the series published in the Journal of American Medical Associations (JAMA) named “The Rational Clinical Examination;” “Evidence-Based Physical Diagnosis” by McGee; “Physical Diagnosis Secrets” by Mangione; and others.

Although it seems that there is a general trend in medical schools in the direction of reducing unnecessary or ineffective physical examination tests, I believe this still needs more attention. Physical examination is a beautiful art that needs to be practiced and refined continuously and presented to students in a meaningful way, especially in this evidence-based medicine era.

### Acknowledgments

This paper was supported by the National Center for Advancing Translational Sciences, National Institutes of Health, through UCSF-CTSI Grant Number UL1 TR001872. Its contents are solely the responsibility of the authors and do not necessarily represent the official views of the NIH.

### Conflict of Interest

The author declares that there are no conflicts of interest.
